# Taxonomy of *Conocybe* I: *Conocybe maculatostriata* and *C. lidaensis* - two new species of *Conocybe* section *Conocybe* (*Bolbitiaceae*, *Agaricales*) from southwestern China

**DOI:** 10.3897/mycokeys.139.192477

**Published:** 2026-08-26

**Authors:** Han-bing Song, Li-yan Tang, Qiao Fang, Song-ming Tang, Zong-long Luo

**Affiliations:** 1 College of Agriculture and Biological Science, Dali University, Dali 671003, Yunnan, China College of Agriculture and Biological Science, Dali University Dali China; 2 Co-Innovation Center for Cangshan Mountain and Erhai Lake Integrated Protection and Green Development of Yunnan Province, Dali University, Dali 671003, Yunnan, China Co-Innovation Center for Cangshan Mountain and Erhai Lake Integrated Protection and Green Development of Yunnan Province, Dali University Dali China

**Keywords:** Morphology, new taxa, phylogenetic placement, section *Conocybe*

## Abstract

In this study, using the phylogenetic framework for *Conocybe* from Tóth et al. and Song, along with a combined dataset of internal transcribed spacer (ITS), nuclear large subunit ribosomal DNA (28S), and translation elongation factor 1-alpha (*tef*1-α) sequences, we reconstructed a Bayesian inference Maximum-likelihood phylogenetic tree for sect. *Conocybe*. The phylogenetic trees showed that sect. *Conocybe* is divided into two monophyletic clades: sect. *Conocybe* Clade 1 is consistent with sect. *Conocybe* in both morphology and phylogenetic positions, whereas some species in sect. *Conocybe* Clade 2 have morphological characteristics that conform to sect. *Mixtae*. Moreover, we also discovered two new species of *Conocybe* in southwestern China: *C. maculatostriata*, which has a pileus with maculate stripes, and *C. lidaensis*, whose caulocystidia and pileocystidia are of a mixed type. In addition, the study provides an identification key for sect. *Conocybe* Clade 1 and 2, accompanied by morphological descriptions and line drawings of *C. mesospora* OTU 21 and the new species.

## Introduction

*Conocybe* Fayod, classified in *Basidiomycota*, *Agaricomycetes*, *Agaricales*, *Bolbitiaceae*, is a genus of small-sized agaricoid fungi with a global distribution (Arnolds 2005; Hausknecht 2009). These fungi are found in diverse habitats, in humus-rich habitats, such as rich pastures, compost and dung heaps, but also in nutrient poor dry grasslands, woodlands, and moist sites (Hausknecht 2009; Song 2025). Using data from GBIF (https://www.gbif.org/) and existing records, Song (2025) showed that species within this group are distributed from temperate to cold regions, a wide ecological range that corresponds to the high taxonomic diversity recorded in global nomenclatural databases. Based on the study by Song (2025), more than 10 species have been recorded in southwestern China.

*Conocybe* originally belonged to the genus *Agaricus* L., Fries in 1821 classified species under tribe *Galera* that possessed a pileus ranging from conical to campanulate (occasionally viscid and lacking a squamulose or pubescent surface), lamellae rust-brown in colour, and a slender stipe with a pruinose or pubescent surface as members of this tribe (Fries 1821). It was not until 1889 that Fayod established the genus *Conocybe* by integrating both macroscopic and microscopic characteristics (Fayod 1889). Subsequently, the taxonomic status and classification of *Conocybe* underwent multiple revisions (Ricken 1915; Atkinson 1918; Kühner 1935; Velenovský 1940; Singer 1951; Kühner and Romagnesi 1953; Moser 1978; Watling 1982; Singer 1986; Bon 1992; Arnolds 2005).

Building upon Singer’s work, Hausknecht and Krisai-Greilhuber (2006) developed a comprehensive infrageneric classification framework for *Conocybe*, which at that time encompassed 10 sections, 13 subsections, and 38 series. This framework gained widespread recognition following the publication of Hausknecht's 2009 monograph, *Conocybe* and *Pholiotina* Fayod in Europe, which primarily focused on European species (Hausknecht 2009). Malysheva (2012a) later established sect. *Heterocystidiae* E.F. Malysheva based on the description and discussion of *C. alkovii* E.F. Malysheva, at which point *Conocybe* was divided into 11 sections, 14 subsections, and 38 series. Tao et al. (2026) treated subsect. *Subdumetorae* E.F. Malysheva as a synonym of subsect. *Dumetorae* Hauskn. & Krisai based on a combined approach of morphology and phylogeny. Consequently, *Conocybe* is now divided into 11 sections, 13 subsections, and 38 series. Within sect. *Conocybe*, Hausknecht and Krisai-Greilhuber (2006) further subdivided the section into eight series. However, the morphological and phylogenetic positions of these eight series are currently inconsistent and difficult to reconcile (Tóth et al. 2013; Song 2025). This incongruence is not limited to the subdivision of sect. *Conocybe* but is also evident in the subdivision of genus *Conocybe* (Song 2025).

In the morphology-based classification system of *Conocybe*, caulocystidia are key characters for the subdivision of the genus (Hausknecht 2009; Song 2025). However, their form can vary at different developmental stages. For instance, as documented by Hausknecht (2009), in *C. subpubescens* P.D. Orton, which belongs to sect. *Mixtae* Singer, caulocystidia are not present on the stipe surface during the primordium stage, whereas lecythiform caulocystidia develop after the basidiomata have matured. In contrast, *C. singeriana* Hauskn., classified under sect. *Pilosellae* Singer, exhibits abundant lecythiform and capilliform caulocystidia in its early stages, which gradually disappear as it matures (Hausknecht 2009). Additionally, within the early-diverging lineage of sect. *Conocybe*, a new species identified in this study features mixed-type caulocystidia and is sister to *C. alkovii*, which possesses lecythiform caulocystidia. Therefore, taxonomic bias can easily arise if the subdivision of *Conocybe* relies solely on caulocystidia, but caulocystidia remain useful diagnostic characters and should be interpreted together with basidiospore morphology, pileipellis structure, pileocystidia, ecology, and molecular phylogenetic evidence. Accordingly, the classification of *Conocybe* and its infrageneric ranks should be evaluated using these additional, more stable morphological characters, a view that is consistent with Hausknecht (2009).

Currently, many species in *Conocybe* lack DNA sequences, or the available sequences for many species are not derived from type specimens (Tóth et al. 2013; Song 2025). However, sequencing of many type specimens currently is underway (International Conocybe consortium, unpubl.) and the situation will be improved in future. For example, the *Conocybe mesospora* s.l. is nested in at least three different clades; *C. mesospora* OTU 21, described in detail in the present study is one of them, but as long as the type specimen of *Conocybe mesospora* Kühner ex Watling is not characterized molecular genetically, no taxonomical consequences can be drawn. In addition, two new species were discovered from southwestern China, enriching the diversity of *Conocybe* sect. *Conocybe* species in this region.

## Materials and methods

### Samplings and morphological analyses

The specimens utilized in this study were collected from Jilin, Fujian, and Yunnan Provinces, as well as the Xizang Autonomous Region, China, between 2023 and 2025. Specimen collection and morphological examination were performed according to the standardized protocols established by Hausknecht (2009), Song and Bau (2025), and Song et al. (2026). The dried specimens are deposited in the Fungarium of Jilin Agricultural University (HMJAU/FJAU).

Microscopic examination involved observing rehydrated tissue sections in distilled water, 5% KOH, or occasionally 1% Congo red. To assess lamellar reactions, a 25% ammonia solution was applied (Hausknecht 2009). Both observation and photomicrography were carried out using a Nikon Eclipse 80i microscope. For macroscopic colors, the RAL color system served as the reference standard (Reichs-Ausschuss für Lieferbedingungen und Gütesicherung, RAL; https://www.ral-guetezeichen.de/). Regarding microscopic measurements (e.g., of basidiospores, basidia, and cystidia), they followed the statistical format detailed in Song and Bau (2025) and Song et al. (2026), in which measurements are expressed as (a)b–c(d), and the notation (n/m/p) denotes the number of spores measured from a given number of basidiomata and collections.

### DNA extraction, PCR amplification, and sequencing

DNA extraction, PCR amplification, and sequencing were carried out according to the protocol described in Song and Bau (2025) and Song et al. (2026). Briefly, total genomic DNA was extracted following the same method. The ITS region was amplified using primers ITS1F/ITS4 (White et al. 1990; Gardes and Bruns 1993), 28S with LR0R/LR7 (Vilgalys and Hester 1990; Moncalvo et al. 2000), and *tef*1-α with EF1-983F/EF1-2218R (Rehner and Buckley 2005). Reaction mixtures and thermal cycling conditions were identical to those in the referenced protocol. Amplification was performed on a Bio-Rad T100™ Thermal Cycler.

PCR products were checked on 1% agarose gels, purified, and sent to Sangon Biotech (Shanghai) Co., Ltd. for sequencing. The ITS region was sequenced in one direction, whereas 28S and *tef*1-α were sequenced bidirectionally. Raw sequence chromatograms were edited using BioEdit v7.2.5 (Hall 1999) with specific trimming: for ITS, the region from the 3’ end of 18S to the 5’ end of 28S was retained; for 28S and *tef*1-α, low-quality ends were removed. Polymorphic sites were coded with IUPAC ambiguity codes. Sequence identities were confirmed by BLAST searches against the NCBI database. The final sequences have been deposited in GenBank (accession numbers provided in Table 1).

**Table 1.** Details of DNA sequences used for phylogenetic reconstruction. Newly generated sequences are shown in bold; missing data are indicated by “–”. ‘T’ denotes the holotype.

**Taxon**	**Voucher specimen**	**GenBank accession numbers**			**Origin**	**References**
		**ITS**	**28S**	***tef*1-α**		
*Bolbitius coprophilus*	HMJAU64958	OQ780315	OQ758216	–	China	Song and Bau 2023
*B. coprophilus*	SZMC-NL-2640	JX968253	JX968370	–	Hungary	Tóth et al. 2013
*B. reticulatus*	WU30001	JX968249	JX968366	JX968455	Hungary	Tóth et al. 2013
*B. subvolvatus*	WU28379	JX968248	JX968365	JX968454	Italy	Tóth et al. 2013
*Candolleomyces leucotephrus*	SZMC-NL-1953	FM163226	FM160683	FM897219	Hungary	Nagy et al. 2011
*Conocybe alkovii*	FJAU71714	PX589399	PX589415	PX610261	China	Song et al. 2026
*C. alticola*	ZRL20240316 T	PQ699270	PQ699294	PQ836632	China	Han et al. 2025
*C. ammophila*	WU23983	JX968197	JX968313	JX968416	Mongolia	Tóth et al. 2013
*C. antipus*	WU19791	JX968215	JX968332	JX968432	Austria	Tóth et al. 2013
*C. aurea*	WU28161	JX968184	JX968300	JX968407	Italy	Tóth et al. 2013
*C. brachypodii*	HMJAU64927	PX746930	PX746938	PX754434	China	Tao et al. 2026
*C. brachypodii*	SZMC-NL-3169	JX968199	JX968316	JX968419	Hungary	Tóth et al. 2013
*C. ceracea*	HMJAU64951 T	OQ758110	OQ758218	OQ758305	China	Song and Bau 2023
*C. coniferarum*	FJAU72632	PX589398	PX589414	PX610260	China	Song et al. 2026
*C. crispella*	WU27367	JX968208	JX968325	JX968426	Australia	Tóth et al. 2013
*C. cylindrospora*	HMJAU42440 T	MG250375	OQ758203	PX610262	China	Liu and Bau 2018; Song and Bau 2023
***C. lidaensis***	**FJAU78854 T**	**PZ058469**	**PZ058475**	**PZ055559**	**China**	**This study**
***C. lidaensis***	**FJAU78855**	**PZ058470**	**PZ058476**	**PZ055560**	**China**	**This study**
*C. deliquescens*	HMJAU61998	OP373403	OQ758204	OQ758292	China	Song and Bau 2023
*C. dumetorum*	SZMC-NL-2693	JX968201	JX968318	JX968421	Sweden	Tóth et al. 2013
*C. echinata*	SZMC-NL-1007	JX968196	JX968312	–	Hungary	Tóth et al. 2013
*C. enderlei*	WU21272	JX968163	JX968279	–	Italy	Tóth et al. 2013
*C. ferruginea*	LAH38015 T	OR773174	OR773178	PV007806	Pakistan	Asif et al. 2025
*C. gigasperma*	SZMC-NL-3972	JX968179	JX968295	JX968403	Slovakia	Tóth et al. 2013
*C. graminis*	WU13466	JX968195	JX968311	–	Austria	Tóth et al. 2013
*C. hausknechtii*	LE253789 T	JQ247194	–	–	Russia	Malysheva 2012b
*C. herbarum*	WU22193	JX968193	JX968309	–	Austria	Tóth et al. 2013
*C. hornana*	SZMC-NL-3499	JX968178	JX968294	JX968402	Slovakia	Tóth et al. 2013
*C. incerta*	LE313017 T	KY614062	–	–	Russia	Malysheva 2017
*C. intrusa*	WU25546	JX968211	JX968328	JX968429	Finland	Tóth et al. 2013
*C. juniana*	SZMC-NL-2105	JX968191	JX968307	JX968413	Hungary	Tóth et al. 2013
*C. leporina*	SZMC-NL-2380	JX968177	JX968293	JX968401	Hungary	Tóth et al. 2013
*C. macrocephala*	WU18148	JX968182	JX968298	–	Austria	Tóth et al. 2013
***C. maculatostriata***	**FJAU78852 T**	**PZ058468**	**PZ058474**	**PZ055558**	**China**	**This study**
***C. maculatostriata***	**FJAU78853**	**PZ058467**	**PZ058473**	**PZ055557**	**China**	**This study**
*C. mandshurica*	LE262844 T	JQ247197	–	–	Russia	Malysheva 2012a
***C. mesospora* OTU 21**	**FJAU78846**	**PZ058471**	**PZ058477**	**PZ055561**	**China**	**This study**
***C. mesospora* OTU 21**	**FJAU78847**	**PZ058472**	**PZ058478**	**PZ055562**	**China**	**This study**
*C. mesospora*	IBL6	MZ410635	–	–	Poland	Direct Submission
*C. mesospora*	GLM 45895	MK412360	AY207173	–	Germany	Direct Submission
*C. mesospora*	FJAU72663	PX746931	PX746939	PX754435	China	Tao et al. 2026
*C. monikae*	WU22612	JX968200	JX968317	JX968420	Austria	Tóth et al. 2013
*C. olivaceopileata*	LE313106 T	KY614059	–	–	Russia	Malysheva 2017
*C. parapilosella*	JLS3063 T	MN872706	–	–	Spain	Siquier and Salom 2020
*C. pilosella*	HMJAU64957	OQ780306	OQ758206	OQ758295	China	Song and Bau 2023
*C. praticola*	HMJAU64965	OQ780303	PX589412	PX610258	China	Song and Bau 2023; Song et al. 2026
*C. pseudocrispa*	HMJAU64944	OQ780308	OQ758211	OQ758298	China	Song and Bau 2023
*C. pubescens*	WU20759	JX968170	JX968286	JX968396	Italy	Tóth et al. 2013
*C. qujingensis*	HKAS127149 T	OQ755415	PP333100	–	China	Lu et al. 2024
*C. qujinguniversitatis*	HKAS128150 T	PP340465	PP333102	–	China	Lu et al. 2024
*C. rickeniana*	SZMC-NL-2468	JX968198	JX968315	JX968418	Hungary	Tóth et al. 2013
*C. rostellata*	SZMC-NL-2499	JX968162	JX968278	JX968390	Sweden	Tóth et al. 2013
*C. rufostipes*	HMJAU64937 T	OQ758120	OQ758227	OQ758317	China	Song and Bau 2023
*C. sabulicola*	WU11185	JX968186	JX968302	JX968409	Italy	Tóth et al. 2013
*C. salalahensis*	NHZ-22-006 T	PP741575	PP741574	PP746605	Oman	Hussain et al. 2025
*C. semiglobata*	WU8794	JX968188	JX968304	JX968168	Austria	Tóth et al. 2013
*C. siennophylla*	HMJAU64966	OQ780312	OQ758210	OQ758297	China	Song and Bau 2023
*C. singeriana*	WU22129	JX968166	JX968282	JX968393	Austria	Tóth et al. 2013
*C. spiculoides*	F36057	MK412347	–	–	Germany	Direct Submission
*C. subovalis*	SZMC-NL-1415	JX968190	JX968306	JX968412	Hungary	Tóth et al. 2013
*C. subxerophytica*	SZMC-NL-0164	JX968187	JX968303	JX968410	Sweden	Tóth et al. 2013
*C. tenera*	SZMC-NL-1615	JX968180	JX968296	JX968404	Hungary	Tóth et al. 2013
*C. volvicystidiata*	LIP0001212 T	KY346827	–	–	France	Hausknecht and Broussal 2016
*C. watlingii*	WU22744	JX968172	JX968288	JX968398	Finland	Tóth et al. 2013
*C. yadongensis*	ZRL20220042 T	PQ699291	PQ699315	PQ836640	China	Han et al. 2025
*Conocybula. coprophila*	HMJAU62008	OR995662	OR995712	PP000855	China	Song and Bau 2024
*Co. cyanopus*	HMJAU62007	OR995663	OR995713	PP000856	China	Song and Bau 2024
*Co. smithii*	HMJAU62001	OP373407	OQ758215	OQ758300	China	Song and Bau 2023; Song and Bau 2024
*Conobolbitina aeruginosa*	WU27104	JX968247	JX968364	–	Germany	Tóth et al. 2013; Song and Bau 2024
*Con. dasypus*	HMJAU62002	OR995661	OR995711	PP000854	China	Song and Bau 2024
*Con. lignicola*	FJAU72922 T	PX612217	PX612214	PX610264	China	Song et al. 2026
*Con. pygmaeoaffinis*	WU16600	JX968149	JX968382	–	Austria	Tóth et al. 2013; Song and Bau 2024
*Descolea antarctica*	NZ5182	AF325647	–	–	USA	Peintner et al. 2001
*D. quercina*	HMJAU64959	OQ780313	OQ758213	OQ758299	China	Song and Bau 2023
*Galerella nigeriensis*	CNF1/5859 T	JX968251	JX968368	JX968457	Nigeria	Tóth et al. 2013
*G. plicatella*	GC-07468	OQ845969	OR039001	–	Italy	Direct Submission
*Pholiotina dentatomarginata*	SZMC-NL-2921	JX968258	JX968374	JX968460	Hungary	Tóth et al. 2013
*P. eburnea*	HMJAU65035 T	OR995694	OR994097	PP000886	China	Song and Bau 2024
*P. intermedia*	HMJAU62014	OR995667	OR995717	PP000860	China	Song and Bau 2024
*P. serrata*	HMJAU62006	OP538570	OQ758217	OQ758301	China	Song and Bau 2023
*P. sulciceps*	HMJAU65100 T	OR995710	OR994113	PP000902	China	Song and Bau 2024
*P. vexans*	HMJAU45078	OR995669	OR995719	PP000862	China	Song and Bau 2024
*Psathyrella piluliformis*	HMJAU37922	MG734716	MW413364	MW411001	China	Yan and Bau 2018

### Sequence alignment and phylogenetic analyses

Phylogenetic analyses were conducted according to the protocols established by Song and Bau (2025) and Song et al. (2026). All sequences utilized in this study were obtained from GenBank (Table 1) and complemented with newly generated data (18 sequences). Multiple alignments of ITS, 28S, and *tef*1-α were generated employing the MAFFT online service (Katoh et al. 2019), subsequently manually optimized in MEGA7, trimming ambiguous regions at the 5’ and 3’ ends. (Kumar et al. 2016), and then assembled into a combined dataset using PhyloSuite v2 (Zhao et al. 2025). The selection of optimal partitioning strategies and nucleotide substitution models was performed with ModelFinder v2.2.0 (Kalyaanamoorthy et al. 2017). Maximum likelihood (ML) phylogenetic estimation was executed in IQ-TREE 3 under 1000 standard bootstrap pseudoreplicates (Nguyen et al. 2015; Wong et al. 2025). Bayesian inference (BI) was implemented in MrBayes v3.2.7a running for one million generations; convergence was confirmed when the average standard deviation of split frequencies fell below 0.01 (final observed value = 0.007) (Ronquist et al. 2012). The phylogenetic trees were visualized and finalized using iTOL (Letunic and Bork 2019), Adobe Photoshop 2021, and Adobe Illustrator 2021. Species from *Psathyrella* (Fr.) Quél. and *Candolleomyces* D. Wächt. & A. Melzer (Song and Bau 2023) were selected as the outgroup.

### Abbreviations

For Latin names: ***B***. = *Bolbitius*; ***C***. = *Conocybe*; ***Ca***. = *Candolleomyces*; ***Co***. = *Conocybula*; ***Con***. = *Conobolbitina*; ***D***. = *Descolea*; ***G***. = *Galerella*; ***P***. = *Pholiotina*; ***Ps***. = *Psathyrella*.

## Results

### Phylogenetic analyses

**Figure 1.**
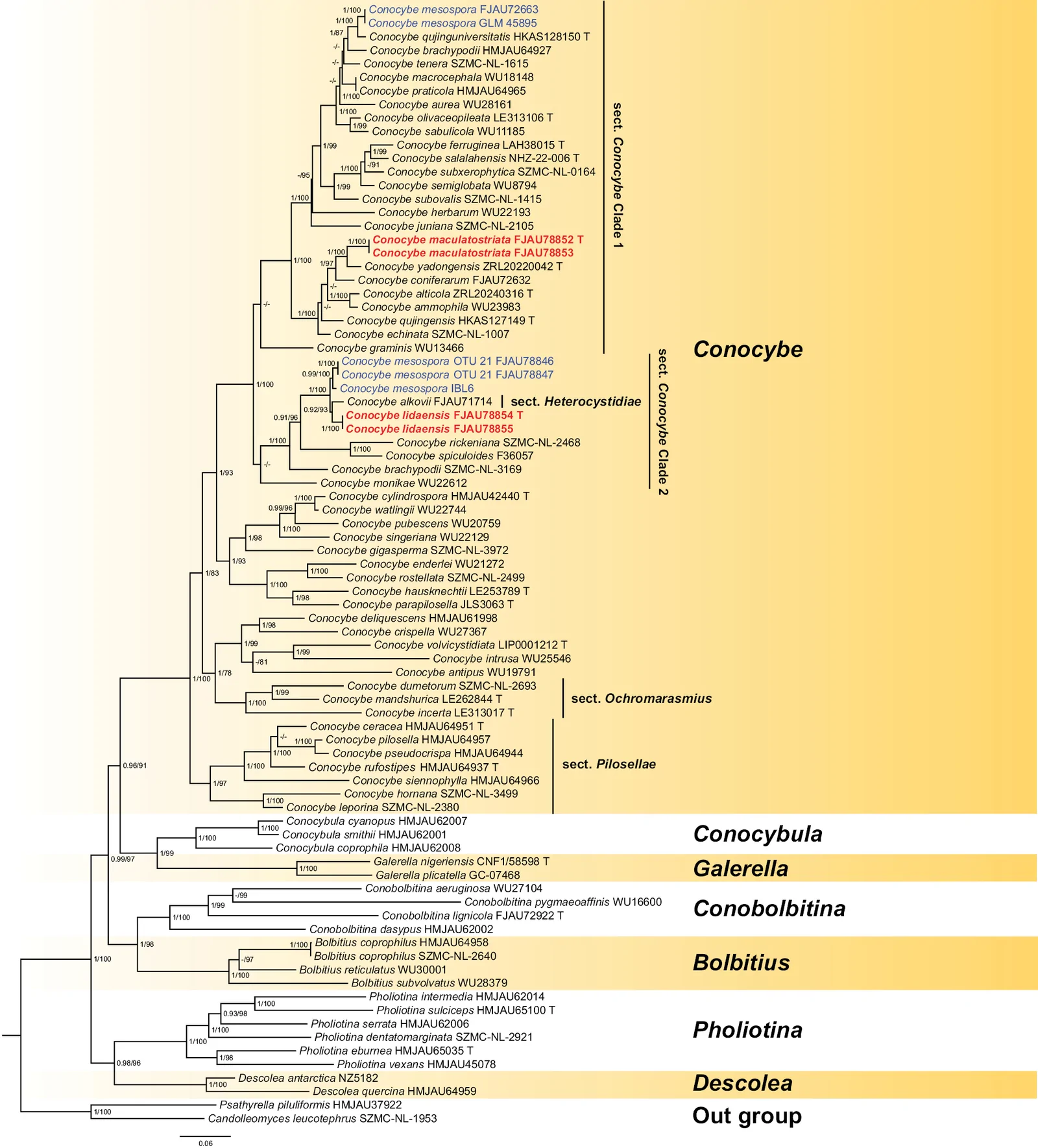
Phylogenetic placement of *Conocybe* sect. *Conocybe* within the *Bolbitiaceae* inferred through Bayesian inference and Maximum likelihood analyses of a combined ITS, 28S, and *tef*1-α dataset. Newly described species proposed in this study are indicated in bold red. The polyphyletic *Conocybe mesospora* is shown in blue. T = holotype.

Phylogenetic reconstruction was performed using both Bayesian inference and Maximum likelihood approaches, based on a concatenated dataset comprising ITS, 28S, and *tef*1-α sequences. The Bayesian tree is shown in Fig. 1 as its topology was identical to that obtained from the ML analysis. Support values, including Bayesian posterior probabilities (PP ≥ 0.90) and ML bootstrap percentages (MLbs ≥ 70%), are indicated at the nodes; nodes failing to meet these thresholds are represented by “–” (Fig. 1).

Sequence alignments yielded 832 bp for ITS, 1,295 bp for 28S, and 1115 bp for *tef*1-α. The concatenated matrix comprised 83 sequences and 3,242 nucleotide positions, among which 1,493 were variable, 1,037 were parsimony-informative, 266 were singleton, and 1,938 were constant. For the ML analysis, optimal substitution models were selected according to the Akaike Information Criterion (AIC): ITS employed GTR+F+R5, 28S employed GTR+F+I+R3, and *tef*1-α employed GTR+I+R5. For the Bayesian analysis, models were selected based on the Bayesian Information Criterion (BIC): GTR+F+I+G4 was applied to both ITS and 28S, while GTR+I+G4 was used for *tef*1-α.

Phylogenetic tree (Fig. 1): The genus *Conocybe* formed a strongly supported monophyletic clade (PP/MLbs = 0.96/91). Within it, sect. *Conocybe* also constituted a highly supported monophyletic lineage (1/93), consisting of two sister clades sect. *Conocybe* Clade 1 and sect. *Conocybe* Clade 2 with full support (1/100).

Within section *Conocybe* Clade 1, specimens FJAU78852 and FJAU78853 form a distinct, maximally supported clade (1/100). This clade is sister to *C. yadongensis* R.L. Zhao & X.X. Han ZRL20220042 with full support (1/100). In sect. *Conocybe* Clade 2, specimens FJAU78854 and FJAU78855 form a maximally supported clade (1/100), which is sister to *C. alkovii* FJAU71714 with support values of 0.92/93.

A BLAST search of the ITS sequence from specimen FJAU78852 against the NCBI database revealed the following similarity percentages: 95.03% to *C. yadongensis* (ZRL20220042), 94.09% to *C. coniferarum* E.F. Malysheva (FJAU72632), 92.43% to *C. alticola* R.L. Zhao & X.X. Han (ZRL20240316), and 91.21% to *C. ammophila* M. Lange (WU23983). For specimen FJAU78854, the ITS sequence showed 97.5% similarity to *C. mesospora* (IBL6), 96.9% to *C. alkovii* (FJAU71714), and 88.27% to *C. rickeniana* (SZMC-NL-2468).

Based on morphological and phylogenetic evidence, we propose two new species: *C. maculatostriata* (represented by specimens FJAU78852 and FJAU78853) and *C. lidaensis* (represented by specimens FJAU78854 and FJAU78855). Morphological evidence is provided in the Taxonomy section.

### Taxonomy

#### Conocybe mesospora

Taxon classificationFungiAgaricalesBolbitiaceae

OTU 21 (according to International Conocybe consortium, unpubl.)

9BC6E411-906C-514F-BE46-FF1C5362A907

**Figure 2.**
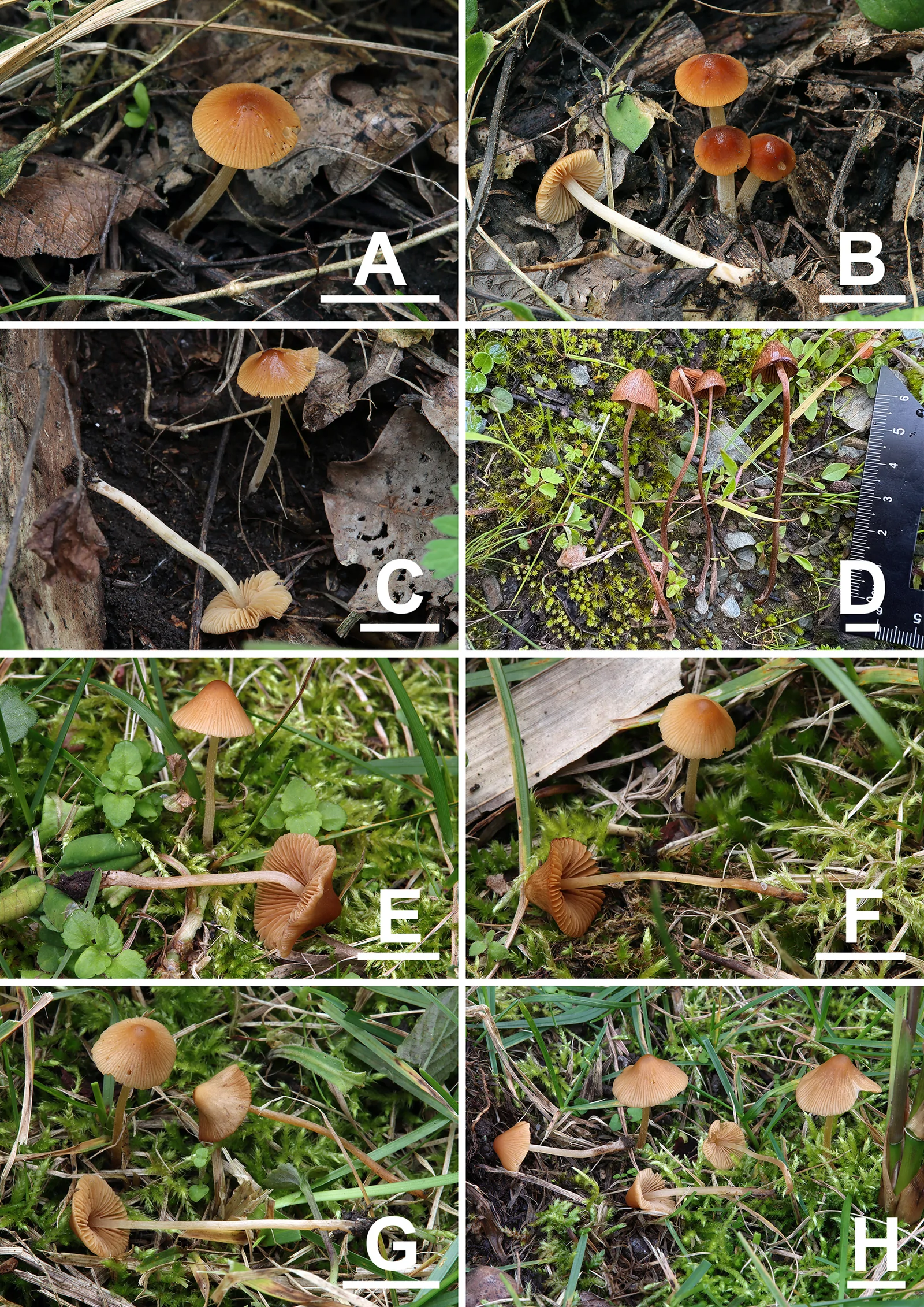
Basidiomata of *Conocybe* species. **A**. *Conocybe mesospora* OTU 21 (FJAU78846); **B**. *C. mesospora* OTU 21 (FJAU78847); **C**. *C. mesospora* OTU 21 (FJAU78849); **D**. *C. maculatostriata* (FJAU78852 T); **E**. *C. lidaensis* (FJAU78854 T); **F**. *C. lidaensis* (FJAU78855); **G**. *C. lidaensis* (FJAU78856); **H**. *C. lidaensis* (FJAU78858). T = holotype. Scale bars: 10 mm.

**Figure 3.**
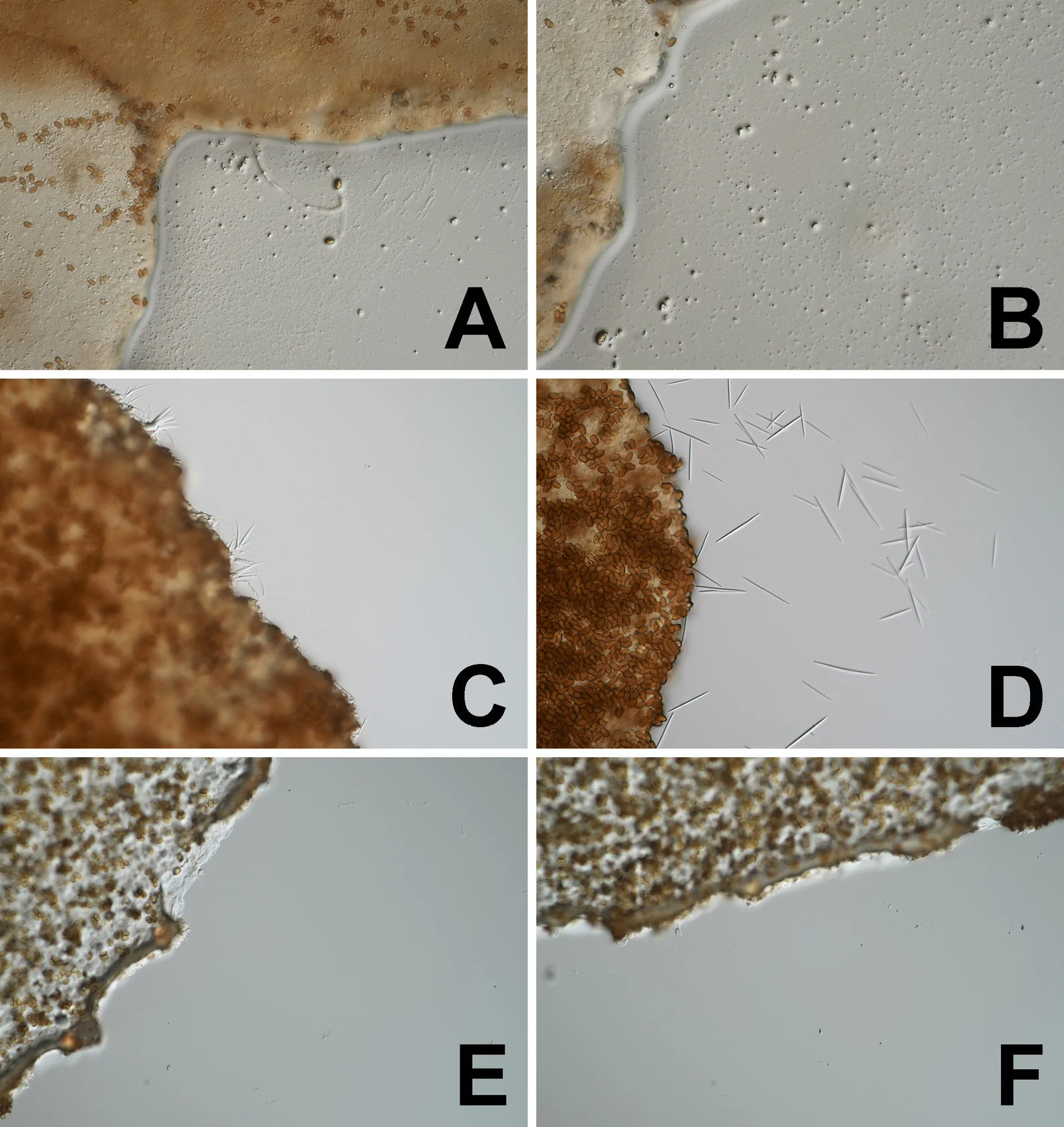
Illustration of the ammonia reaction in *Conocybe* species. **A, B**. *Conocybe mesospora* OTU 21 (FJAU78846); **C, D**. *C. maculatostriata* (FJAU78852 T); **E, F**. *C. lidaensis* (FJAU78854 T). T = holotype.

**Figure 4.**
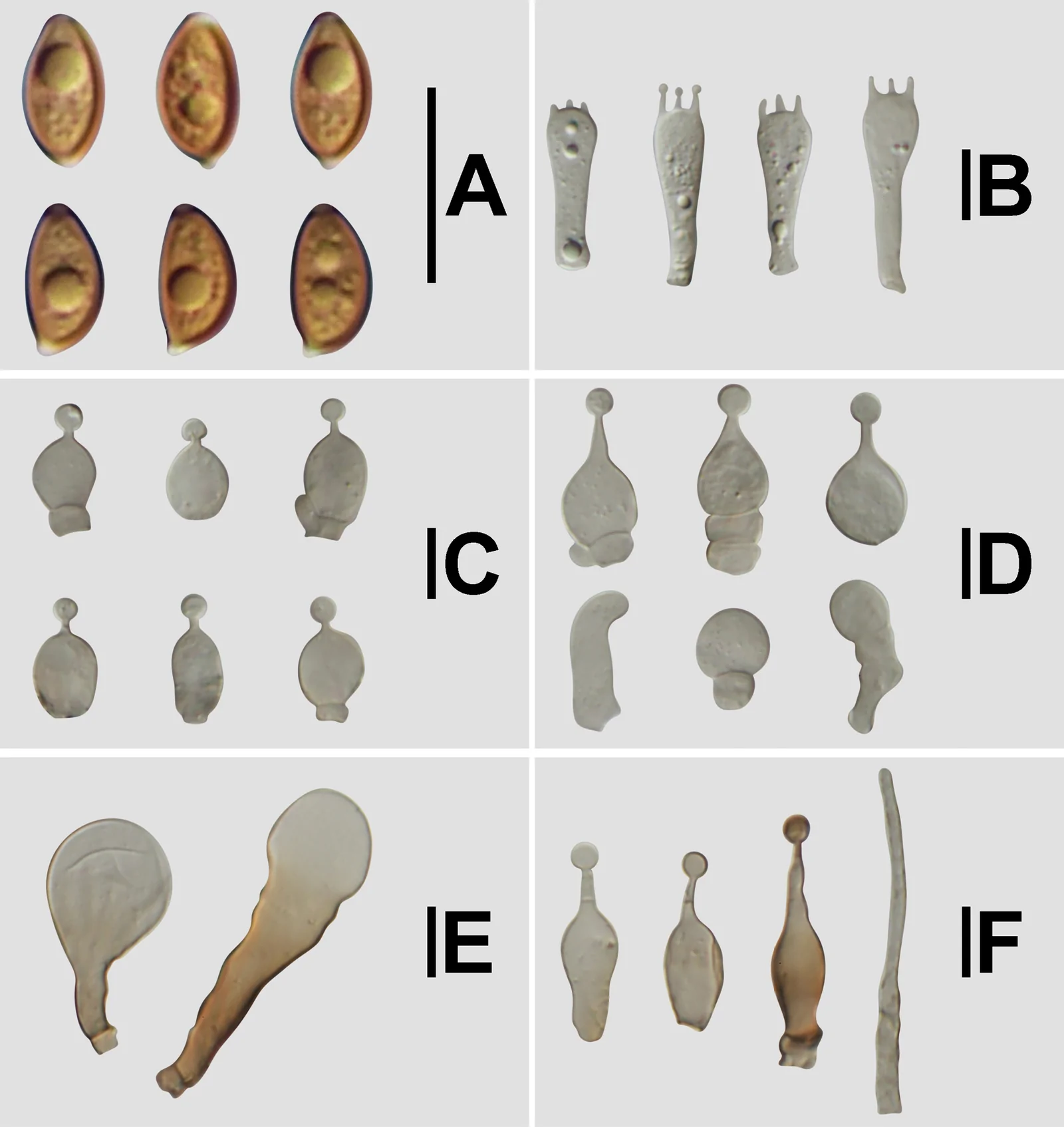
Microscopic structures of *Conocybe mesospora* OTU 21 (FJAU78846). **A**. Basidiospores; **B**. Basidia; **C**. Cheilocystidia; **D**. Caulocystidia; **E**. Pileipellis elements; **F**. Pileocystidia. Scale bars: 10 μm.

**Figure 5.**
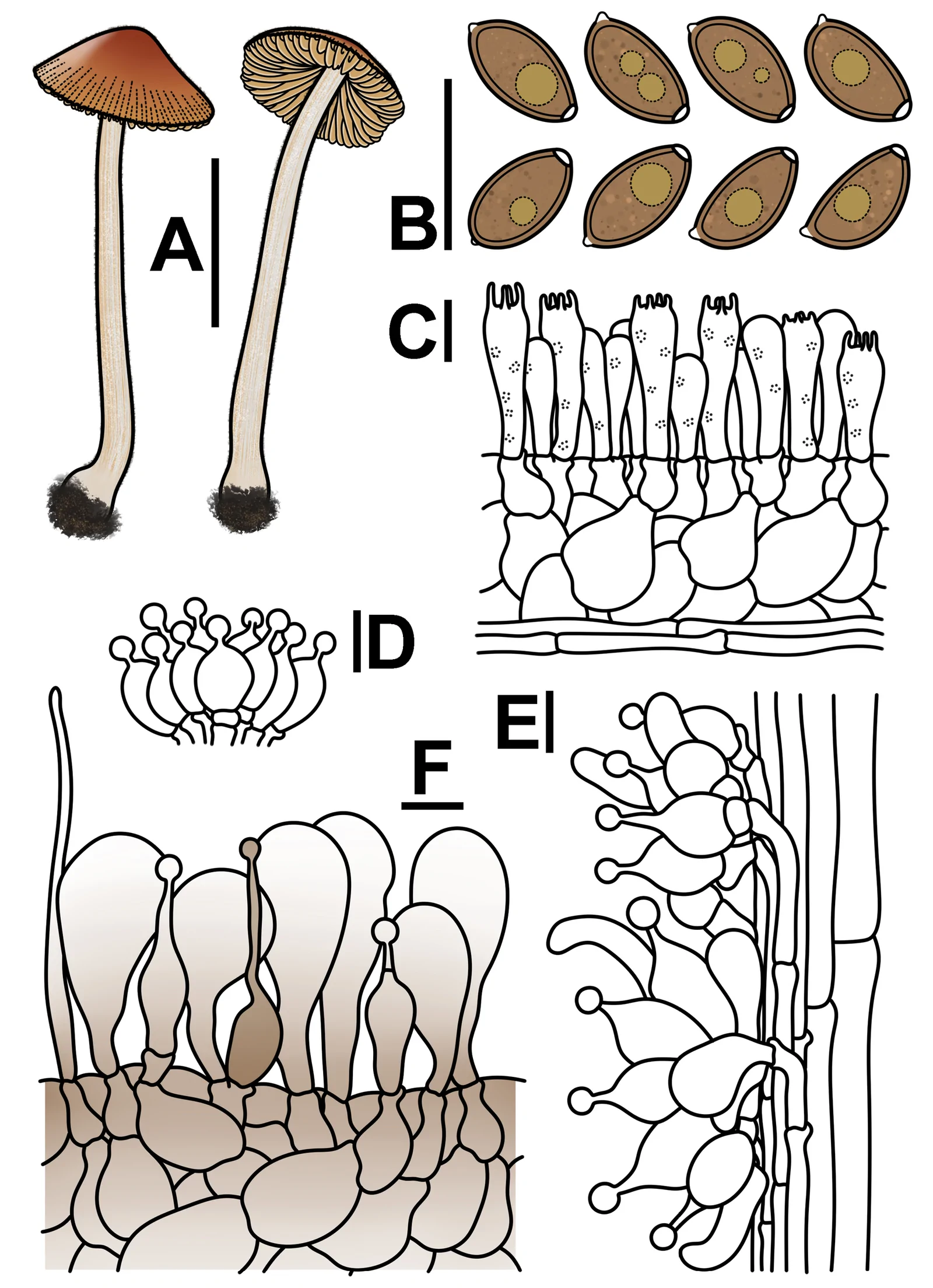
*Conocybe mesospora* OTU 21 (FJAU78846) **A**. Basidiomata; **B**. Basidiospores in 5% KOH solution; **C**. Hymenium and subhymenium; **D**. Cheilocystidia; **E**. Stipitipellis; **F**. Pileipellis. Scale bars: 10 mm (**A**); 10 μm (**B–F**).

Figs 2A–C, 3A, 3B, 4, 5

##### Description.

***Basidiomata*** mycenoid. ***Pileus*** 5–15 mm diam, initially paraboloid to obtusely conical, later broadly conical, subumbonate, margin aspects straight, undulate. At the young stage, the center ranges from red-orange (RAL 2001) to traffic orange (RAL 2009) and bright red-orange (RAL 2008), while the margin varies from saffron yellow (RAL 1017) to pastel yellow (RAL 1034) and ochre brown (RAL 8001), the colors become darker, with the pileus center changing from red-brown (RAL 8012) to pearl orange (RAL 2013), and the margin transitioning from pastel yellow (RAL 1034) to ochre brown (RAL 8001) at maturity. Hygrophanous, smooth, slightly viscid, pubescent, striate up to half center. ***Context*** thin, pastel yellow (RAL 1034) to ochre brown (RAL 8001), no distinct odor or taste. ***Lamellae*** adnexed to narrowly adnate, ventricose, moderately crowded, unequal, sand yellow (RAL 1002) to ivory (RAL 1014), pastel yellow (RAL 1034), margin even to slightly eroded. ***Stipe*** 15–25 mm long, 1–2 mm thick, cylindrical, light ivory (RAL 1015) to ivory (RAL 1014), surface pruinose and pubescent, with longitudinal fibrillose striate, base subbulbous.

***Basidiospores*** (60/3/3) (6.8–)7.3–8.3(–8.7) × 3.8–4.7 μm, Q = (1.65–)1.69–2.02(–2.1), Q_m_ = 1.82(±0.09), non-lentiform, oblong to subcylindrical, slightly amygdaliform, thick walled, containing oil droplets, germ pore approximately 1–2 μm in diameter, ochre brown (RAL 8001) to clay brown (RAL 8003) in KOH solution. ***Basidia*** (19–)21–29(–30) × 7–9 μm, narrowly clavate to clavate, 4-spored, sterigmata 2–5 μm long, vacuolar contents present. ***Cheilocystidia*** 15–20(–23) × (6–)7–11 μm, lecythiform (body lageniform, broadly ellipsoid to oblong, some subglobose), with capitula 3–4 μm wide. ***Pleurocystidia*** absent. ***Caulocystidia*** (16–)18–26(–29) × (7–)8–13 μm, predominantly lecythiform (ca. 90%, body lageniform, broadly ellipsoid to oblong, some subglobose), capitula 3–6 μm wide, intermixed with clavate, subglobose and subellipsoid elements. ***Pileipellis*** epithelioid hymeniderm, composed of spheropedunculate and clavate cells (27–)29–43(–45) × (10–)12–18 μm, basal region with fawn brown (RAL 8007) pigment. ***Pileocystidia*** (25–)26–34(–38) × (6–)7–12 μm, lecythiform to tibiiform (ca. 90%, body lageniform, broadly ellipsoid to oblong), capitula 3–5 μm wide, some pigmented in water and KOH solution, occasionally with filamentous elements. All structures with clamp connections. Negative reaction with ammonia.

##### Habitat.

The specimens collected in this study grow solitary or gregarious on the humus layer near *Sambucus williamsii* Hance during summer and autumn.

##### Known distribution.

Jilin Province, Siping City, China.

##### Additional collection examined.

China • Jilin Province, Siping City, Yitong Manchu Autonomous County, 18 August 2025, 43°36'00"N, 125°12'11"E, alt. 369 m, L.J. Zhao, S25081913 (FJAU78846), S25081914 (FJAU78847). China • Jilin Province, Siping City, Yitong Manchu Autonomous County, 23 August 2025, 43°36'00"N, 125°12'11"E, alt. 369 m, H.B. Song S25082309 (FJAU78848). China • Jilin Province, Siping City, Yitong Manchu Autonomous County, 25 August 2025, 43°35'54"N, 125°12'11"E, alt. 387 m, H.B. Song S25082519 (FJAU78849). China • Jilin Province, Siping City, Yitong Manchu Autonomous County, 29 September 2025, 43°35'17"N, 125°11'57"E, alt. 457 m, H.B. Song S25092901 (FJAU78850), S25092903 (FJAU78851). Sequences were obtained from all specimens mentioned above, and their morphology was also examined.

##### Notes.

The description of *Conocybe mesospora* OTU 21 was originally intended to clarify the phylogenetic position of *C. mesospora*. However, *C. mesospora* lacks a type sequence, and available non-type sequences are polyphyletic (*C. mesospora* IBL6 and *C. mesospora* GLM 45895) and in at least three different clades (located in sect. *Conocybe* Clade 1 and Clade 2). Morphological evidence alone is insufficient to support a definite taxonomic conclusion, and the sequence of the material is within the *C. mesospora* OTU 21 of the International Conocybe consortium (unpubl.). To decide which one of the currently differentiated OTUs is in accordance with the type of *C. mesospora* will require sequences obtained from the French type material (Singer and Hausknecht 1992; Hausknecht 2009).

#### Conocybe maculatostriata

Taxon classificationFungiAgaricalesBolbitiaceae

H.B. Song & S.M. Tang sp. nov.

39B4DFBD-C037-5217-A06B-2F3251C98D49

862176

**Figure 6.**
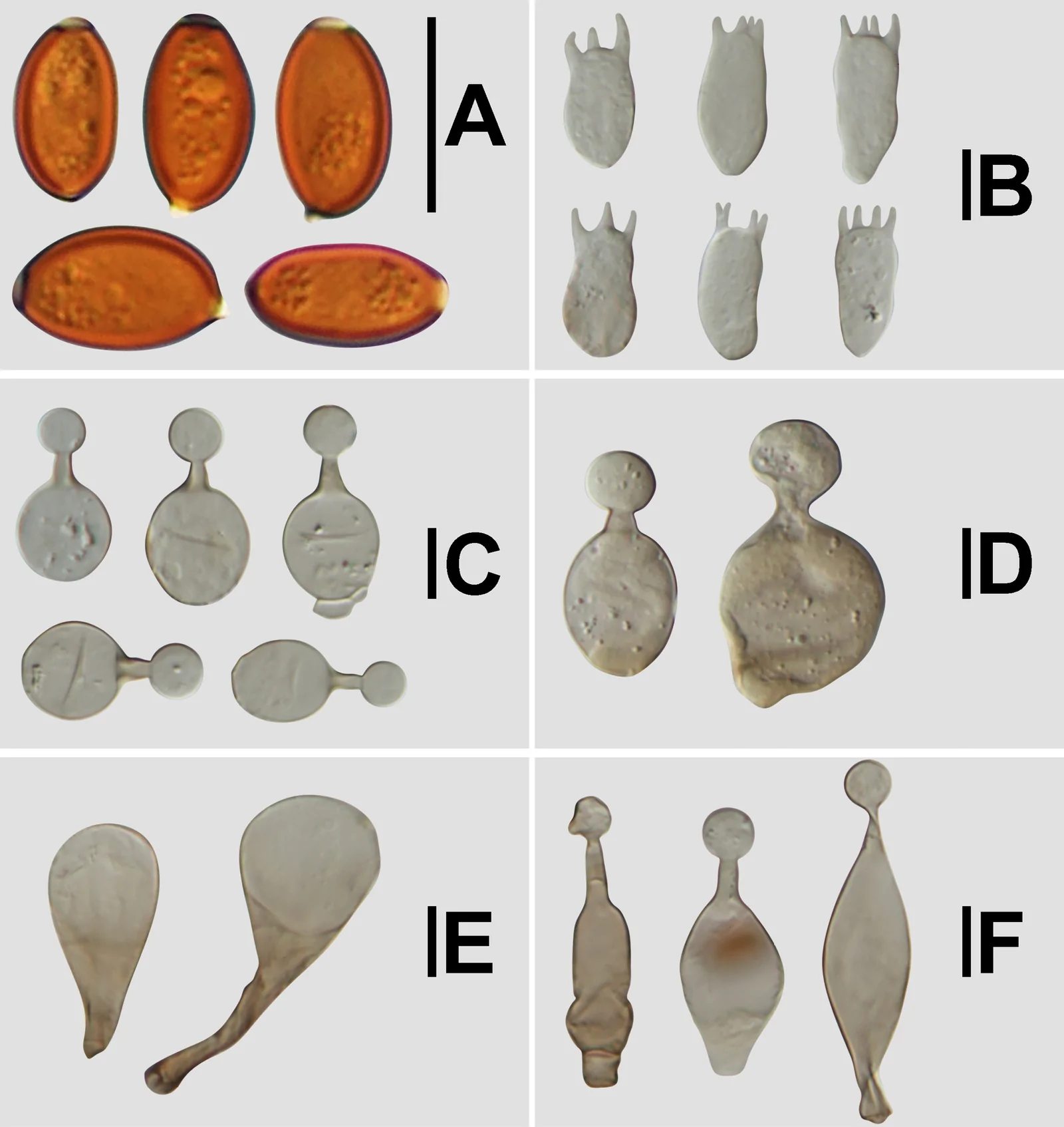
Microscopic structures of *Conocybe maculatostriata* (FJAU78852). **A**. Basidiospores; **B**. Basidia; **C**. Cheilocystidia; **D**. Caulocystidia; **E**. Pileipellis elements; **F**. Pileocystidia. Scale bars: 10 μm.

**Figure 7.**
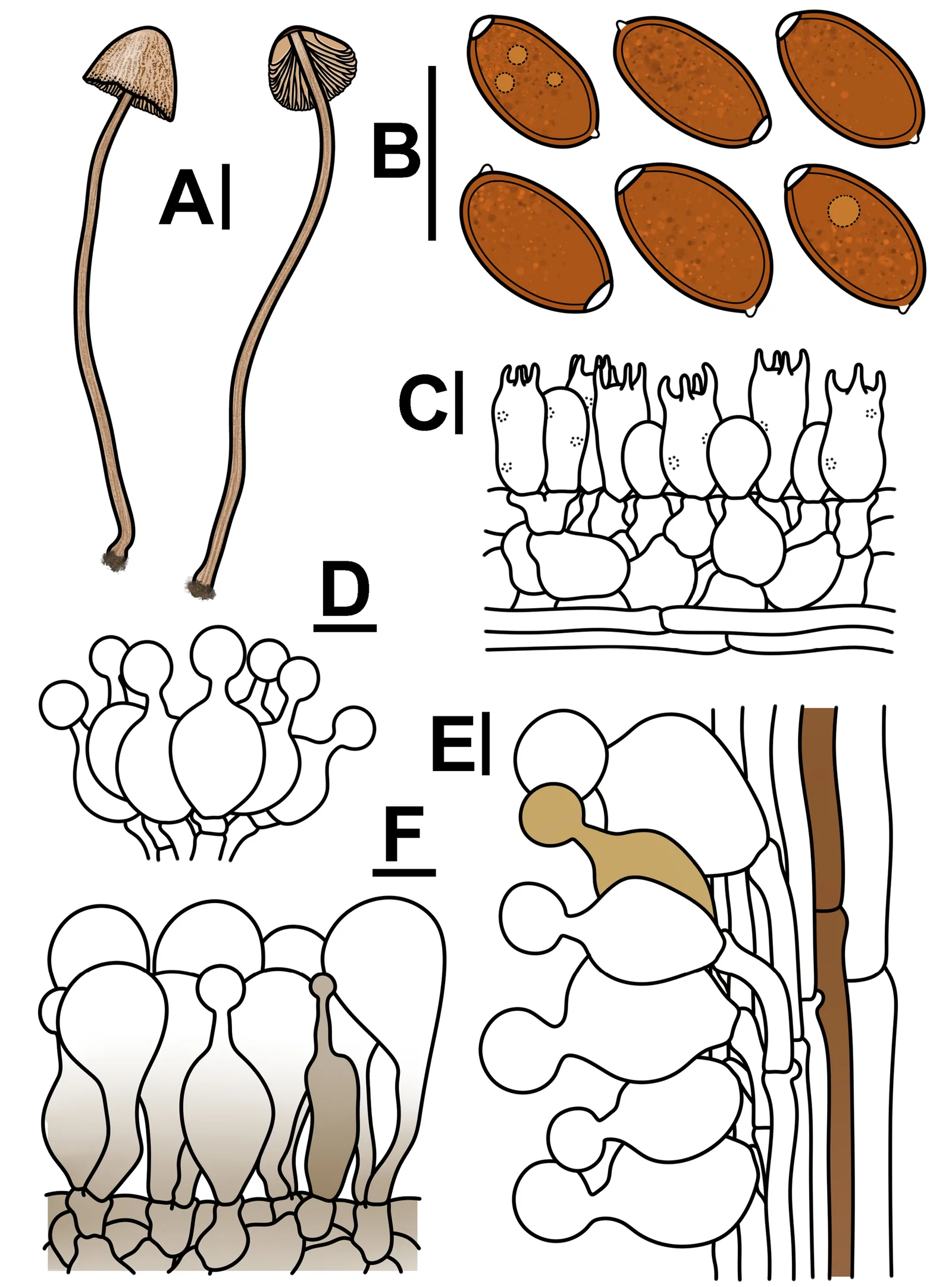
*Conocybe maculatostriata* (FJAU78852). **A**. Basidiomata; **B**. Basidiospores in 5% KOH solution; **C**. Hymenium and subhymenium; **D**. Cheilocystidia; **E**. Stipitipellis; **F**. Pileipellis. Scale bars: 10 mm (**A**); 10 μm (**B–F**).

Figs 2D, 3B, 3C, 6, 7

##### Etymology.

The specific epithet “maculatostriata” refers to a pileus with maculate stripes when moist.

##### Holotypus.

China • Xizang Autonomous Region, Xigazê City, Chomo County, 24 July 2025, 27°37'12"N, 88°56'24"E, alt. 3607 m, S.M. Tang, 2025072431 (FJAU78852).

##### Diagnosis.

The key taxonomic feature of this species is its pileus with maculate stripes when moist and the large capitulum diameter of its lecythiform cystidia; the latter feature overlaps with species of ser. *Magnicapitata*. However, it can be distinguished from other species in ser. *Magnicapitata* by its maculate stripes when moist, slightly larger basidiospores (9.7–12.8 μm), and the median constriction observed in some basidia.

##### Description.

***Basidiomata*** mycenoid. ***Pileus*** 5–15 mm, first nearly hemispherical to obtusely conical, then paraboloid to campanulate, aspects of margin straight, undulate, center clay brown (RAL 8003) to copper brown (RAL 8004), salmon pink (RAL 3022), margin salmon pink (RAL 3022) to beige red (RAL 3012), copper brown (RAL 8004). Hygrophanous, maculate stripes when moist, smooth, glabrous, striate to half the pileus center. ***Context*** thin, salmon pink (RAL 3022) to copper brown (RAL 8004), with no specific odor or taste. ***Lamellae*** adnexed to narrowly adnate, ventricose to nearly segmentiform, moderately crowded, unequal in length, ochre brown (RAL 8001) to copper brown (RAL 8004), orange brown (RAL 8023), margin even. ***Stipe*** 10–25 mm long, 0.5–1 mm thick, cylindrical, salmon pink (RAL 3022) to copper brown (RAL 8004), red brown (RAL 8012), surface pruinose, with longitudinal fibrillose striations, base subbulbous.

***Basidiospores*** (60/3/2) (9.2–)9.7–12.8(–13.1) × (5.1–)5.3–7.2(–8) μm, Q = (1.44–)1.49–2.03(–2.16), Qm=1.77(±0.18), lateral view slightly ellipsoid to oblong, subcylindrical, occasionally frontal view faintly hexagonal, wall slightly thick, containing oil droplets, germ pore diameter 1.5–2.5 μm, basidiospores in KOH solution copper brown (RAL 8004) to pearl orange (RAL 2013). ***Basidia*** (16–)17–24(–27) × 8–12 μm, polymorphic, clavate to cylindrical, some median constriction, utriform, 4-spored, sterigmata 2–5 μm long, vacuolar contents in basidia. ***Cheilocystidia*** (22–)23–30(–31) × (8–)10–15(–16) μm, lecythiform (body lageniform, broadly ellipsoid to oblong, some subglobose), capitula 6–9 μm wide. ***Pleurocystidia*** absent. ***Caulocystidia*** (23–)28–44(–48) × 15–26(–27) μm, all are lecythiform (body lageniform, broadly ellipsoid to oblong, some subglobose), capitula 7–16 μm wide. ***Pileipellis*** epithelioid hymeniderm, composed of spheropedunculate and clavate cells (23–)27–48(–53) × (11–)12–24 μm, basal pigments clay brown (RAL 8003) to olive brown (RAL 8008). ***Pileocystidia*** (29–)32–53(–54) × (5–)7–13(–15) μm, all are lecythiform to tibiiform (body lageniform, broadly ellipsoid to oblong, partially fusiform to cylindrical), capitula 4–8 μm wide, some pigment-filled. All structures with clamp connections. Weakly positive reaction to ammonia, forming needle-shaped crystals.

##### Habitat.

Scattered growth on plateau grasslands in summer.

##### Known distribution.

Xizang Autonomous Region, Xigazê City, China.

##### Additional collection examined.

China • Xizang Autonomous Region, Xigazê City, Chomo County, 24 July 2025, 27°37'12"N, 88°56'24"E, alt. 3607 m, S.M. Tang, 2025072428 (FJAU78853).

##### Notes.

Based on the presence of exclusively lecythiform caulocystidia, *C. maculatostriata* belongs to sect. *Conocybe*. Within this section, its assignment to the ser. *Magnicapitata* Hauskn. & Krisai is justified by the notably large capitula of its lecythiform cystidia, which, based on existing species descriptions, range from 4–18 μm (Hausknecht and Krisai-Greilhuber 2006). *Conocybe maculatostriata* differs from *C. spiculoides* Kühner & Watling in ser. *Magnicapitata*, as the latter lacks a germ pore on its basidiospores and has basidiospores less than 9.5 μm long (Watling 1980). It is distinguished from *C. echinata* (Velen.) Singer and *C. rickeniana* P.D. Orton, as both have notably shorter basidiospores (average length < 10 μm) and a negative reaction to ammonia (Orton 1960; Singer 1989). *Conocybe maculatostriata* differs from *C. proxima* Singer in that the latter has basidiospores shorter than 9 μm (6.8–8.6 μm) and grows on decaying wood (Singer and Digilio 1951). The new species can be distinguished from *C. juniana* (Velen.) Hauskn. & Svrček, inclusive of its var. *sordescens* (P.D. Orton) Hauskn. & Svrček, and var. *subsejuncta* Hauskn. by the latter indistinctly striate pileus and rugose pileus surface (Hausknecht 2009). *Conocybe maculatostriata* differs from *C. amazonica* Singer in that the latter has 13.5–15.5 μm long basidiospores, and grows on wood blocks in the Amazon rainforest (Singer 1989). Phylogenetically, *C. maculatostriata* is closely related to *C. coniferarum* and *C. yadongensis* R.L. Zhao & X.X. Han. However, *C. coniferarum* has slightly smaller, 8.5–10 μm, lentiform basidiospores, a negative reaction to ammonia, and grows in coniferous forests, while *C. yadongensis* has basidiospores shorter than 10 μm (8.4–9.1 μm), and a brownish orange to yellowish brown pileus without maculate stripes (Malysheva 2017; Han et al. 2025).

#### Conocybe lidaensis

Taxon classificationFungiAgaricalesBolbitiaceae

H.B. Song & Z.L. Luo sp. nov.

FC191718-1E68-526D-9610-C2141C60BA40

862177

**Figure 8.**
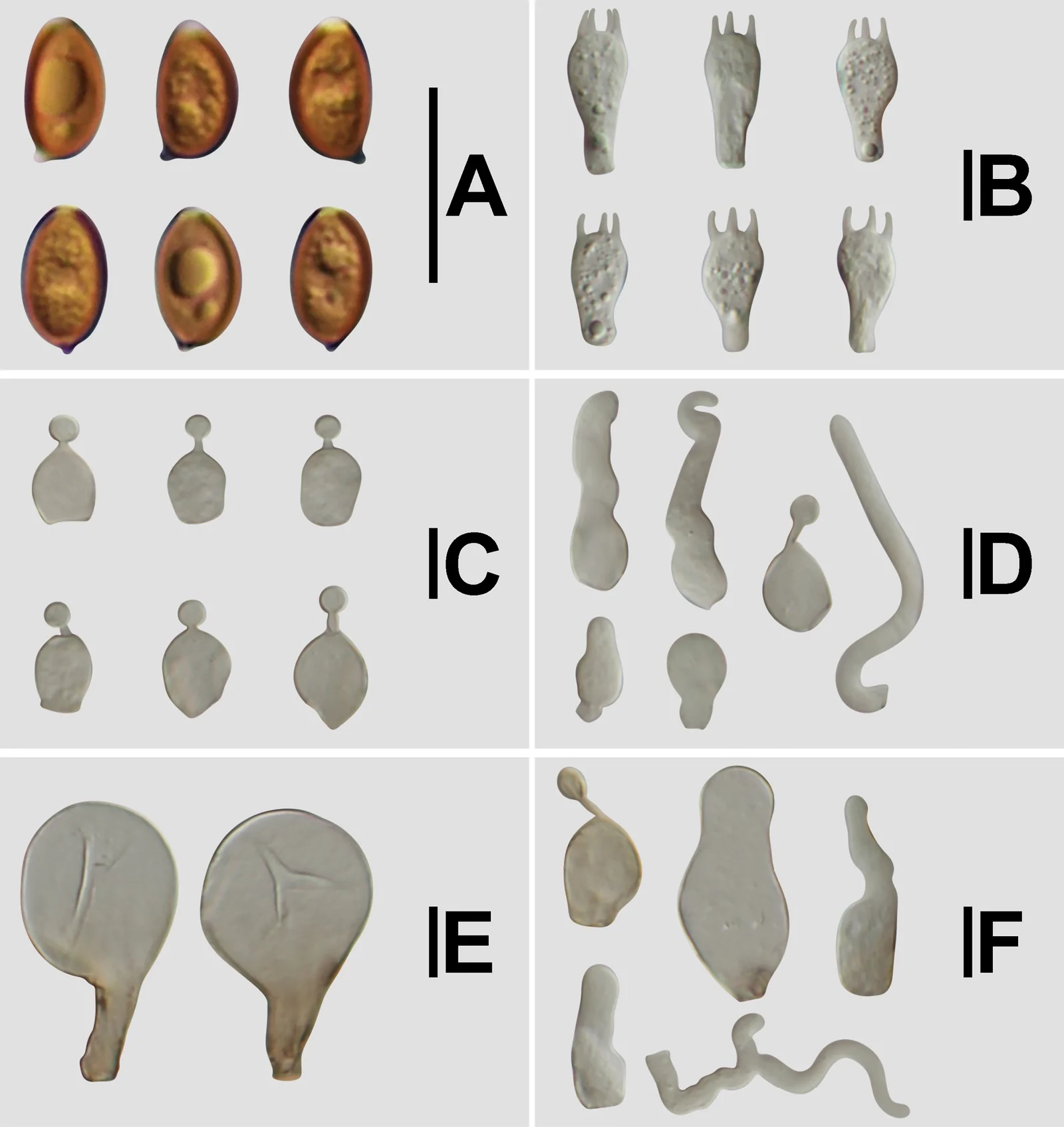
Microscopic structures of *Conocybe lidaensis* (FJAU78854). **A**. Basidiospores; **B**. Basidia; **C**. Cheilocystidia; **D**. Caulocystidia; **E**. Pileipellis elements; **F**. Pileocystidia. Scale bars: 10 μm.

**Figure 9.**
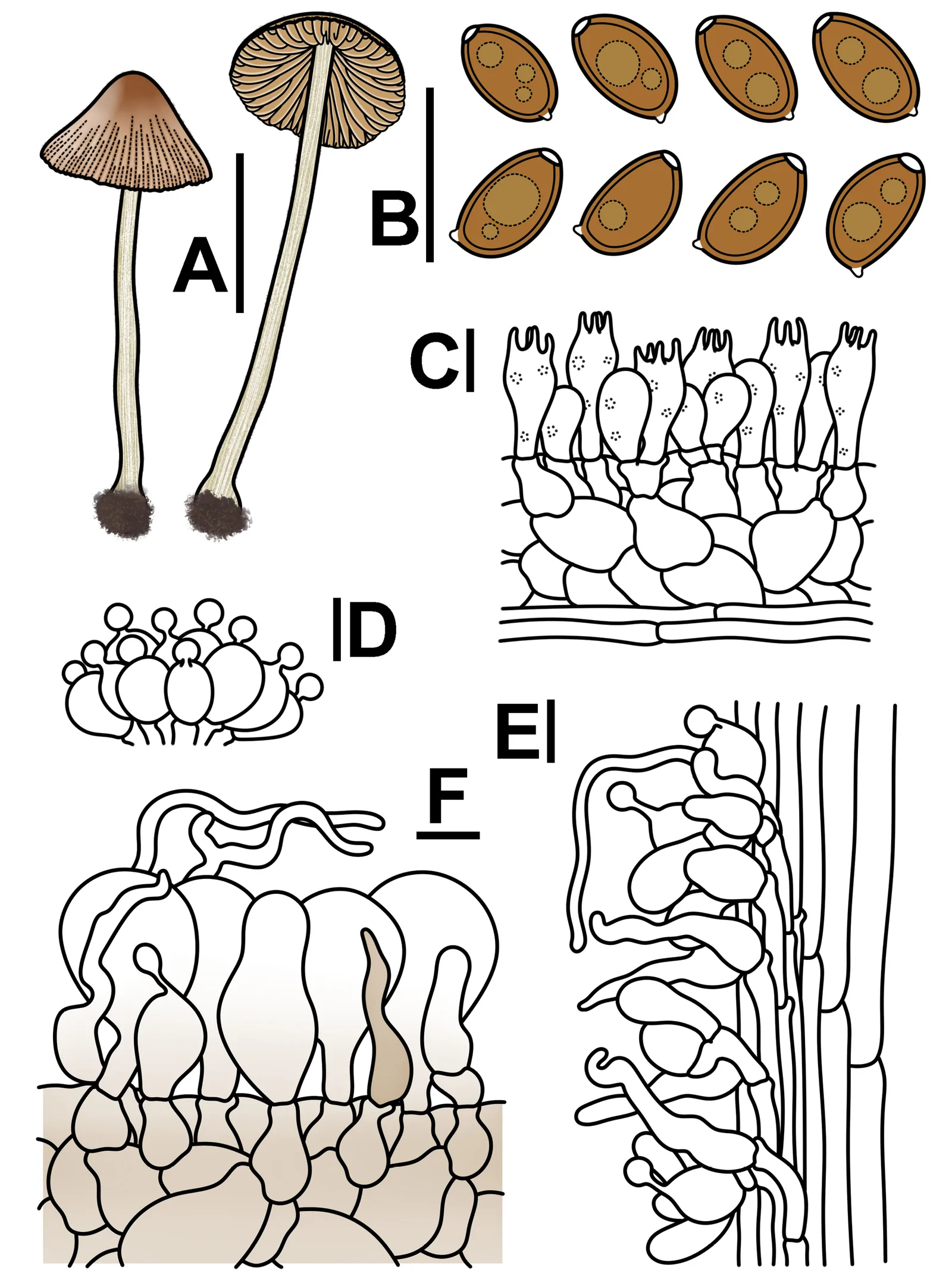
*Conocybe lidaensis* (FJAU78854). **A**. Basidiomata; **B**. Basidiospores in 5% KOH solution; **C**. Hymenium and subhymenium; **D**. Cheilocystidia; **E**. Stipitipellis; **F**. Pileipellis. Scale bar: 10 mm (**A**); 10 μm (**B–F**).

Figs 2E, 2F, 2G, 2H, 3E, 3F, 8, 9

##### Etymology.

The specific epithet “lidaensis” refers to the type locality, Dali University (abbreviated as “lida”), where the holotype of this species was collected.

##### Holotypus.

China • Yunnan Province, Dali Bai Autonomous Prefecture, Dali City, Dali University, 25 October 2025, 25°40'22"N, 100°09'18"E, alt. 2177 m, H.B. Song, S25102503 (FJAU78854).

##### Diagnosis.

*Conocybe lidaensis* may be confused with mature *C. mesospora* s.l. but lacks the bright orange pileus color; *C. lidaensis* further differs by a higher percentage of mixed type caulo- and pileocystidia, with lecythiform cystidia in less than 40% of them, whereas in *C. mesospora* lecythiform cystidia are dominant and other shapes are rare. *Conocybe lidaensis* grows in moss layers in bamboo forest grasslands. It also shows distinct molecular differences, genetically.

##### Description.

***Basidiomata*** mycenoid. ***Pileus*** diameter 5–20 mm, initially conical to obtusely conical, later slightly campanulate to broadly conical, subumbonate, margin aspects straight, undulate. Fresh and moist pileus center ochre brown (RAL 8001) to clay brown (RAL 8003), olive brown (RAL 8008), margin beige (RAL 1001) to ivory (RAL 1014); with age pileus color deepens, clay brown (RAL 8003), chestnut brown (RAL 8015) to mahogany brown (RAL 8016). Hygrophanous, smooth, pubescent (under a lens), striate up to 4/5 of pileus center. ***Context*** thin, same color as pileus, with no specific odor or taste. ***Lamellae*** adnexed to narrowly adnate, ventricose, crowded, unequal in length, light ivory (RAL 1015) to ivory (RAL 1014), brown beige (RAL 1011) to ochre brown (RAL 8001), margin even to slightly eroded. ***Stipe*** 10–40 mm, thickness 0.5–2 mm, cylindrical, light ivory (RAL 1015) to ivory (RAL 1014), khaki grey (RAL 7008), surface with pruinose and short pubescent, showing longitudinal fibrillose-like striate, base subbulbous.

***Basidiospores*** (60/3/3) (6.6–)6.8–7.8(–8) × 4.1–4.7(–4.9) μm, Q = (1.52–) 1.55–1.79(–1.92), Qm = 1.66(±0.08), slightly ellipsoid to oblong, ovoid, somewhat amygdaliform, wall thick, germ pore diameter about 0.5–1.5 μm, ochre brown (RAL 8001) to clay brown (RAL 8003) in KOH solution. ***Basidia*** (14–)15–21(–23) × 7–10 μm, clavate, 4-spored, sterigmata length 3–5 μm, with vacuolar contents in basidia. ***Cheilocystidia*** (15–)16–21(–22) × 7–11 μm, lecythiform (body lageniform, broadly ellipsoid to oblong, some subglobose), with capitula 3–5 μm wide. ***Pleurocystidia*** absent. ***Caulocystidia*** 9–35(–39) × (5–)6–11(–14) μm, mixed, lecythiform (less than 40%, body lageniform, broadly ellipsoid to oblong, some subglobose), with capitula 4–6 μm wide, mixed with ellipsoid, clavate, utriform, lageniform to long-necked lageniform, neck flexuose, occasional capilliform elements, up to 60 μm. ***Pileipellis*** epithelioid hymeniderm, composed of spheropedunculate cells (25–)26–49(–51) × 18–30(–32) μm, with fawn brown (RAL 8007) pigment at the base. ***Pileocystidia*** 21–36 × 5–16 μm, mixed type, lecythiform (ca. 30%, body lageniform, broadly ellipsoid to oblong), with capitula 3–5 μm wide, some filled with pigment, intermixed with utriform, lageniform, long-necked lageniform, and capilliform elements. All structures with clamp connections. Negative reaction with ammonia.

##### Habitat.

In autumn, scattered mossy grassland in bamboo forests.

##### Known distribution.

Yunnan Province, Fujian Province, China.

##### Additional material examined.

China • Yunnan Province, Dali Bai Autonomous Prefecture, Dali City, Dali University, 25 October 2025, 25°40'22"N, 100°09'18"E, alt. 2177 m, H.B. Song, S25102504 (FJAU78855), S25102505 (FJAU78856), S25102506 (FJAU78857), S25102507 (FJAU78858). China • Fujian Province, Sanming City, 9 September 2023, J.M. Liao, LJP9900 (FJAU78859). Sequences were obtained from all specimens mentioned above, and their morphology was also examined.

##### Notes.

*Conocybe lidaensis* is characterized by the presence of mixed types caulo- and pileocystidia, both of which contain lecythiform cystidia. Based on morphological criteria, *C. lidaensis* would be placed in sect. *Mixtae* (Singer 1962; Hausknecht 2003). However, phylogenetically, it is positioned at the base of sect. *Conocybe*, where it is sister to *C. alkovii* within sect. *Heterocystidiae* (Hausknecht and Krisai-Greilhuber 2006; Malysheva 2012a). *Conocybe lidaensis* can be distinguished from other species in sect. *Conocybe* by its mixed caulocystidia (Hausknecht 2009). Additionally, its pileocystidia comprise a combination of lecythiform, utriform, lageniform to long-necked lageniform cystidia and capilliform elements, which differentiates it from species in sect. *Mixtae*. In the closely related *C. alkovii* the caulocystidia in accordance with sect. *Conocybe* and the applanate pileus allow clear morphological distinction between the two taxa (Malysheva 2012a).

### Key to Chinese species of sect. *Conocybe* Clade 1 and 2

**Table:** 

1	Pileus brightly colored	**2**
–	Pileus dull-colored	**3**
2	Pileus apricot-yellow	***Conocybe aurea***
–	Pileus red-orange	***C. mesospora* OTU 21**
3	Pileus surface pubescent	**4**
–	Pileus surface glabrous	**8**
4	Caulocystidia lecythiform and non-lecythiform mixed	***C. lidaensis***
–	Caulocystidia are predominantly lecythiform	**5**
5	Pileipellis without capilliform elements	***C. alticola***
–	Pileipellis with capilliform elements	**6**
6	Stipe pruinose and distinctly pubescent	***C. praticola***
–	Stipe pruinose only and glabrous	**7**
7	Pileus applanate or slightly depressed	***C. alkovii***
–	Pileus obtusely conical to hemispherical	***C. tenera***
8	Pileocystidia absent	***C. yadongensis***
–	Pileocystidia presence	**9**
9	Basidiospores lentiform	**10**
–	Basidiospores not lentiform	**12**
10	Caulocystidia apical diameter 4–6 μm	***C. semiglobata***
–	Caulocystidia apical diameter greater than 6 μm	**11**
11	Exclusive to coniferous forests	***C. coniferarum***
–	Occurring in mixed forests or grasslands	***C. qujingensis***
12	Caulocystidia apical diameter 7–16 μm	***C. maculatostriata***
–	Caulocystidia apical diameter less than 7 μm	**13**
13	Basidiospores longer than 9 μm	***C. brachypodii***
–	Basidiospores shorter than 9 μm	***C. qujinguniversitatis***

## Discussion

Based on the phylogenetic framework proposed by Tóth et al. (2013) and Song (2025), the phylogeny of *Conocybe* sect. *Conocybe* was reconstructed using a concatenated dataset of ITS, 28S, and *tef*1-α gene sequences. The results indicate that sect. *Conocybe* comprises two independent evolutionary lineages, namely sect. *Conocybe* Clade 1 and sect. *Conocybe* Clade 2. Of these, sect. *Conocybe* Clade 1 exhibits morphological characters fully consistent with the definition of sect. *Conocybe*, and its phylogenetic position is consistent; whereas sect. *Conocybe* Clade 2 exhibits transitional features intermediate between sect. *Mixtae* and sect. *Conocybe* (Singer 1962; Hausknecht and Krisai-Greilhuber 2006).

In phylogenetic analyses, the non-type sequences of *C. mesospora* are polyphyletic. Several *C. mesospora* OTUs are distinguished by the International Conocybe consortium (unpubl.). However, as long as the type specimen is not checked, molecularly, biologically or epitypified, the exact phylogenetic position of *C. mesospora* remains open. One of our specimens, which is in accordance with *C. mesospora* OTU 21 (Int. Conocybe consortium, unpubl.), is described in detail here providing habitat photographs, microstructural images, line drawings, and molecular sequence data, so that the issue can be dealt with further in future.

Within sect. *Conocybe* Clade 1, we discovered a new species, *C. maculatostriata*, collected from Chomo County, Xigazê City, Xizang Autonomous Region. Based on morphological characters, this species is assigned to ser. *Magnicapitata* (Hausknecht and Krisai-Greilhuber 2006). Among the species closely related to this new species, *C. yadongensis* has cheilocystidia with 5.3–7.2 μm wide capitula, which largely conforms to the definition of ser. *Magnicapitata*; unfortunately, its original description does not include information on caulocystidia (Han et al. 2025). The morphological characters of *C. coniferarum* fully match the definition of ser. *Magnicapitata* (Malysheva 2017). In contrast, the morphological characters of *C. alticola* do not conform to the definition of ser. *Magnicapitata*, and its description similarly lacks information on caulocystidia (Han et al. 2025). Additionally, based on its original description, another related species, *C. ammophila*, clearly does not belong to this series (Hausknecht et al. 2004). Therefore, based on currently available data, the phylogenetic position of ser. *Magnicapitata* cannot be determined with certainty.

Within sect. *Conocybe* Clade 2, we also discovered a new species, *C. lidaensis*, collected from Dali University, Dali City, Dali Bai Autonomous Prefecture, Yunnan Province. This species is sister to *C. alkovii* in sect. *Heterocystidiae* (Malysheva 2012a), but the average basidiospore length of *C. alkovii* is 7.8–8.6 μm. The caulocystidia of *C. lidaensis* and *C. monikae* Hauskn. are consistent with sect. *Mixtae*, but pileocystidia are absent in *C. monikae*, whereas the remaining species exhibit caulocystidia typical for sect. *Conocybe* (Hausknecht and Krisai-Greilhuber 2003; Hausknecht 2009). Therefore, *C. lidaensis* may represent a transitional taxon.

As for the new species described in this paper, their significance extends beyond simply demonstrating species diversity. They also raise important systematic questions, such as how to deal with sect. *Conocybe* Clade 2, which exhibits morphological characteristics of both sect. *Conocybe* and sect. *Mixtae*? We consider that if sect. *Conocybe* Clade 2 possesses shared morphological characters that distinguish it from sect. *Conocybe* and sect. *Mixtae*, it would be possible to consider revising the circumscription of the formerly monotypic sect. *Heterocystidiae* and expanding it to include all species of sect. *Conocybe* Clade 2 (Hausknecht 2009; Malysheva 2012a). However, at present we have specimens of only three species within sect. *Conocybe* Clade 2, which is insufficient to identify stable distinguishing characters for this clade. Additional specimens and sequence data for the other species in this clade will be required to further clarify this issue and refine the taxonomic delimitation of sect. *Conocybe*. International collaboration—for example with the Conocybe Consortium—is necessary, as that group already holds numerous sequences from type material and from additional collections, and a comprehensive revision of the genus is forthcoming.

## Supplementary Material

XML Treatment for Conocybe mesospora

XML Treatment for Conocybe maculatostriata

XML Treatment for Conocybe lidaensis

## Additional information

### Artificial Intelligence (AI) use

The authors accept full responsibility for the content of the manuscript, including the disclosure of any use of AI.

No AI tools were used in the preparation of this manuscript.

### Author ORCIDs

H.-b. Song https://orcid.org/0000-0002-7440-5444

S.-m. Tang https://orcid.org/0000-0003-0665-6900

Z.-l. Luo https://orcid.org/0000-0001-7307-4885

### Data availability

All the sequences have been deposited in GenBank (https://www.ncbi.nlm.nih.gov), and the nomenclatural novelties (new species names) have been registered in MycoBank (https://www.mycobank.org). The data presented in this study are deposited in the Zenodo repository, accession number https://doi.org/10.5281/zenodo.18795077.
